# Pubic bone osteomyelitis outcomes in patients with malignancies: a case series from an academic cancer center

**DOI:** 10.5194/jbji-10-571-2025

**Published:** 2025-12-09

**Authors:** Alexander M. Lewis, Max Vaynrub, Peter A. Mead, Melanie Betchen, Mini Kamboj, Anna Kaltsas

**Affiliations:** 1 Molecular Pharmacology Program, Sloan Kettering Institute, Memorial Sloan Kettering Cancer Center, New York, NY, USA; 2 The Warren Alpert Medical School of Brown University, Providence, RI 02912, USA; 3 Orthopaedic Surgery Service, Department of Surgery, Memorial Sloan Kettering Cancer Center, 1275 York Avenue, New York, NY, USA; 4 Infectious Disease, Department of Medicine, Memorial Sloan Kettering Cancer Center, New York, NY, USA; 5 Department of Medicine, Weill Cornell Medical College, New York, NY, USA

## Abstract

**Introduction**: Pubic bone osteomyelitis (PBO) is a rare complication with sometimes delayed development in patients who have received radiotherapy or surgery of the pelvic region for cancer treatment. Treatment options range from antibiotics alone to pubic bone debridement and source control via diversion of gastrointestinal (GI) or genitourinary (GU) tract fistulae. In this single-center case series of patients with cancer, we sought to characterize outcomes of PBO. **Methods**: We conducted a retrospective analysis of 26 patients, admitted for PBO to Memorial Sloan Kettering Cancer Center between 2017 and 2024. Demographic, clinical presentation, microbiology, treatment, and outcome data were evaluated. Patients were followed until date of death or date of last follow-up. **Results**: Of the 26 patients, 23 were male (88 %) and 3 were female (12 %), with a median age at diagnosis of 70.5 years. The median follow-up period was 680 d. 18/26 (69 %) had fistulas to the pubic bone. 15 patients (58 %) received antibiotics alone. 11 patients (42 %) underwent pubic bone debridement; 8 underwent additional GI or GU diversion procedures for source control. In the group who received surgery, 9/11 (81 %) were ambulating without assistive devices at end of follow-up. In those receiving antibiotics alone, 9/15 (60 %) died a median of 466 d from diagnosis of PBO. **Conclusion**: In our case series, a combination of surgical debridement plus targeted antibiotic therapy offered the best outcomes. However, some patients achieved improvement in symptoms with antibiotic management alone when more aggressive surgical interventions were not feasible.

## 1 Introduction

Osteomyelitis of the pubic symphysis is a rare infectious inflammatory condition that makes up less than 1 % of all osteomyelitis cases, and, if left untreated, it can lead to significant long-term morbidity and, rarely, become potentially life threatening (Ross and Hu, 2003; Peltola and Pääkkönen, 2014; Wechsler et al., 2021). Risk factors for pubic bone osteomyelitis (PBO) include current or past pelvic malignancy, osteoradionecrosis from pelvic radiation, and urological or other pelvic surgery (Wechsler et al., 2021; Hansen et al., 2022). Tumor- or surgery-related anatomic disruptions can form genitourinary (GU) or gastrointestinal (GI) fistulas and lead to microbial seeding of the pubic bones and progressive infection. Radiographically, PBO manifests as bone edema, cortical erosion, abscess formation, and septic arthritis of the pubic symphysis. The presentation of PBO typically involves pain in the anterior pubis, but patients can also experience pain in other areas of the pelvis, including groin, upper thighs, abdomen, hips, or lower back, usually accompanied by difficulty in walking, which may become progressive and irreversible (Ross and Hu, 2003; Pham and Scott, 2007; Hawkins et al., 2015; Shu et al., 2021).

Given the relative rarity of this infection, there are no guidelines to suggest best management practices for this entity specifically. Even less data exist on best practices in patients who are unable to undergo aggressive surgeries for source control and debridement of infected bone, due to underlying co-morbidities or poor overall health status, as is often the case in patients with cancer. Currently published society guidelines focus only on vertebral osteomyelitis (Berbari et al., 2015) or speak more generically to pyogenic osteomyelitis but not specifically to the management of pubic bone osteomyelitis (Spellberg et al., 2022).

Best practices for PBO management are crucial to aid in early diagnosis, attempt curative treatment and improve outcomes, preserve mobility, and ensure optimal symptom alleviation (Upadhyayula, 2020; Wechsler et al., 2021). Due to its subtle and insidious onset, PBO diagnosis is often delayed. Failure to identify and treat PBO early in its pathogenesis can result in ongoing bone and joint destruction, compromising the integrity of the pubic symphysis and pelvic structures, potentially leading to pelvic ring instability, impaired load transfer, sacral insufficiency fractures, and irreversible joint and ligamentous damage (Sexton et al., 1993; Gupta et al., 2015; Chen et al., 2021; Devlieger et al., 2021; Sambri et al., 2021).

In addition, there is a paucity of literature on the treatment of PBO specifically in people with cancer who may have unique risk factors related to surgical or tumor-related anatomic predisposition, radiation, and chemotherapy. Due to the complex nature of the infection, pubic bone debridement, the mainstay of osteomyelitis management, may not always be feasible or safe (Romanò et al., 2010). On the other hand, if only antibiotics are used for treatment, microbial eradication may not be achieved (Smeyers et al., 2024). Therefore, the clinical characterization of the risks and outcomes of this rare bone infection with myriad presentations is essential to provide the best outcomes for patients and minimize long-term morbidity (Upadhyayula, 2020; Wechsler et al., 2021).

In the present retrospective case series, we sought to evaluate outcomes of PBO in a cancer patient population. We performed a retrospective case series of 26 consecutive patients treated at Memorial Sloan Kettering Cancer Center from 2017 to 2024.

## 2 Methods

We retrospectively studied all patients who had an infectious disease consult requested for pubic bone osteomyelitis (PBO) at our institution between January 2017 and December 2024, by querying the infectious disease consult database. PBO was defined by its presence on X-ray, CT, or MRI as interpreted by the reading radiologist as probable osteomyelitis using the terms “bony destruction,” and “pubic symphysis cortical erosion or diastasis” or by chart review when an orthopedic surgeon or infectious disease specialist also made the clinical diagnosis of PBO based on imaging, physical exam findings, and culture results. Demographic data were extracted from charts, including age of diagnosis, sex, co-morbidities, smoking status, cancer diagnoses, prior cancer-related treatment including radiation, date of last radiation, previous urethral manipulations, the presence of indwelling urinary hardware at PBO diagnosis, previous pelvic surgery, symptoms on presentation, and the days from the last cancer treatment (radiotherapy, chemotherapy, or surgery) to PBO diagnosis. Treatment variables were evaluated, including whether an interventional radiologist acquired a bone biopsy or aspiration, whether surgical debridement was performed, and the type and duration of antibiotic therapy.

History of radiation therapy involved any radiation to the abdominal or pelvic area prior to PBO diagnosis. Timing of pelvic radiation relative to date of PBO diagnosis was also recorded.

Tissue, blood, and bone culture patient samples were tracked from the date closest to diagnosis, and cultures that were positive were used to guide antibiotic management.

Patients in the *antibiotics-only group* were defined by the absence of orthopedic surgical intervention (pubic bone debridement and/or addressing any fistulae) for the treatment of PBO, except for obtaining bone or tissue biopsies for cultures.

Patients in the pubic bone *debridement group* underwent surgical intervention as defined by pubic bone debridement in addition to antibiotic administration, and in cases where they also received additional urinary or colorectal diversion procedures, this was also recorded.

Patients were followed through date of death or date of last follow-up at our institution. Treatment outcomes were defined as ambulating with or without assistance at time of last follow-up at our institution and whether or not they were described as having any pubic-bone-related pain (pain in the pelvis, in the groin, or radiating down the legs) in clinical notes at the time of last follow-up. General outcomes such as whether there was progression of pubic bony destruction on last imaging prior to death or last follow-up was tracked, and mortality status was also recorded.

Descriptive statistics were performed by describing the number of patients in each statistical category with a percentage of their respective group sample and by calculating the median and interquartile range (IQR).

## 3 Results

Between January 2017 and December 2024, 26 patients met the search criteria for PBO; among these, 23 were males (88 %) and 3 were females (12 %). The median (interquartile range (IQR)) for the age of diagnosis was 70.5 (12.8), with a range of 34 to 85 years old. Underlying cancer, co-morbid conditions, and other clinical characteristics at presentation are shown in Table 1.

**Table 1:** Patient characteristics, diagnostic details, symptoms at presentation, and treatment.

Age of diagnosis, median (IQR)	70.5 (12.8)
Male, n (%)	23 (88)
Female, n (%)	3 (12)
Cancer types, n (%) (note: some patients had more than one concurrent cancer)	
Prostate	18 (69)
Bladder	5 (19)
Rectal	2 (7)
Anal	1 (4)
Vaginal	1 (4)
Uterine	1 (4)
Sarcoma	1 (4)
Intramuscular myxoma	1 (4)
Gastric mixed tumor	1 (4)
Smoking history, n (%)	
Never smoker	10 (38)
Former smoker	13 (50)
Active smoker	3 (12)
Diabetes, n (%)	9 (35)
History of any radiation therapy, n (%)	20 (77)
Previous urethral manipulation, n (%)	22 (85)
Previous pelvic surgery, n (%)	23 (88)
Presence of any indwelling urinary hardware at diagnosis, n (%)	12 (46)
MRI for diagnosis, n (%)	20 (77)
CT for diagnosis, n (%)	12 (46)
Destruction or erosion of symphysis on imaging, n (%)	26 (100)
Pelvic radiation received > 1 year from diagnosis date ( n= 20), n (%)	16 (80)
Days after last cancer treatment to PBO diagnosis, median (IQR)	713 (2426)
Fever at diagnosis ( n= 25), n (%)	6 (24)
Septic arthritis of the pubic symphysis, n (%)	8 (31)
Genitourinary or gastrointestinal fistula, n (%)	18 (69)
Abscess, n (%)	20 (77)
C-reactive protein (mg dL^−1^), ( n= 17), median (IQR)	4.72 (7)
Erythrocyte sedimentation rate ≥ 30 (mm h^−1^) ( n= 16), n (%)	14 (88)
White blood count ( × 10^9^ L^−1^), median (IQR)	10.4 (5.2)
Microbiology data	
Blood culture taken, n (%)	16 (62)
Blood culture positive ( n= 16), n (%)	0 (0)
Bone culture taken, n (%)	19 (73)
Bone culture positive ( n= 19), n (%)	12 (63)

**Table 1:** Continued.

Treatment, n (%)	
Patients treated with antibiotics only, n (%)	15 (58)
Antibiotics-only patients with pubic bone biopsy taken for culture, n= 15, n (%)	12 (80)
Surgical management	11 (42)
Resection/debridement of the pubic symphysis, n= 11, n (%)	8 (73)
Debridement with concurrent gastrointestinal or urinary diversion, n= 11, n (%)	8 (73)
Antibiotic treatment duration (days), median (IQR)	6.5 (3.8)
Days from admission to discharge, median (IQR)	17 (65)
Pain relief at end of antibiotics, n (%)	
Antibiotic-only treatment group, n= 15, n (%)	8 (53)
Surgical debridement treatment group, n= 11, n (%)	9 (82)
Ambulation at 6-week follow-up, n= 24, n (%)	23 (96)
Antibiotic-only treatment group, n= 14, n (%)	13 (93)
Surgical debridement treatment group, n= 10, n (%)	10 (100)
Patient alive at end of follow-up, n (%)	16 (61)
Antibiotic-only treatment group, n= 15, n (%)	6 (40)
Surgical debridement treatment group, n= 11, n (%)	10 (91)

Imaging modalities used for PBO diagnosis included MRI for 14 patients (54 %), CT for 5 patients (19 %), MRI and CT for 6 patients (23 %), and CT and X-ray for 1 patient (4 %). All patients had destruction or erosion of the pubic bone and/or symphysis by imaging. Most patients (18/26, 69 %) had either a genitourinary (GU) or gastrointestinal (GI) fistula interacting with the pubic symphysis on imaging, and 20 (77 %) had an abscess.

All patients had at least one cancer diagnosis, the most common being either prostate (54 %) or bladder cancer (8 %), with one patient each having non-genitourinary-related cancers, including rectal, leiomyosarcoma, anal, uterine, and vaginal. Some patients had more than one cancer. 20 patients (77 %) had received previous radiation therapy to the pelvic area, and 23 of the 26 (88 %) had previous pelvic surgeries, including genitourinary tract procedures such as stents. More than 80 % of those who had a history of pelvic radiation had received it more than 1 year prior to PBO diagnosis. Notably, there was a large date range from the date of last cancer-directed therapy to the date of PBO diagnosis, with a range of 50 to 7244 d (median: 460 d). In one patient, it was nearly 20 years from the date of last cancer-directed therapy that they presented with pubic bone osteomyelitis.

In terms of presenting symptoms, the most common symptoms included, in 96 % of patients, difficulty ambulating and chronic and/or excruciating pain of the pubic area, suprapubic area, pelvis, hips, back, abdomen, groin, buttocks, or perineum. Fever was uncommon. In a single case, PBO was asymptomatic and diagnosed incidentally on CT imaging, followed by MRI and culture results.

**Figure 1:**
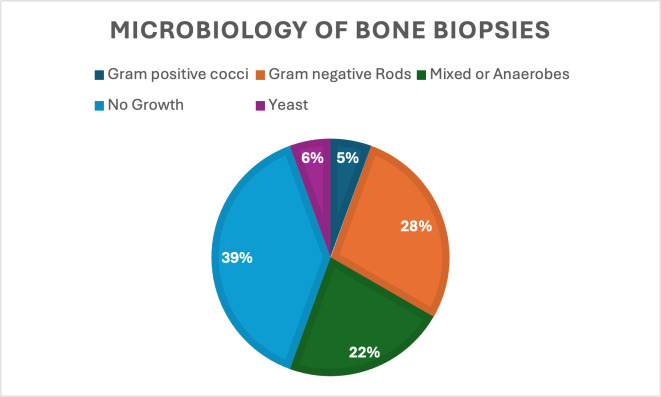
Microbiology of bone biopsies.

In terms of laboratory findings at time of diagnosis, the median (IQR) value for CRP at the date closest to PBO diagnosis of 17 patients was 4.72 mg dL^−1^ (7), and WBC was 10.4 × 10^9^ L^−1^ (5.2) on the date of diagnosis. Of 16 patients where ESR data were available, an erythrocyte sedimentation rate ≥ 30 mm h^−1^ was present in 14 patients (88 %). None of these patients had bacteremia. Of the 19 who did have bone biopsies, 12 had either a positive gram stain or growth in cultures for a 63 % positivity rate, and in 11 cases an organism was isolated in culture (Table 2). Target amplicon sequencing was not pursued in any of the cases. Of the 7 patients who had no growth in bone biopsies, 3 of them were already on antibiotics at the time of biopsy. There were 3 patients whose bone cultures grew organisms despite being on antibiotics at the time of biopsy as well. Aggregate microbiology results are displayed in Fig. 1 and Table 2.

**Table 2:** Microbiology of bone biopsies, n= 19.

Culture type	n (%)
No growth	7 (39) – note: 3 of these were done while the patient was on antibiotics
Gram-negative rods	5 (28)
Mixed growth or anaerobes	4 (22)
Yeast	1 (6)
Gram-positive cocci	1 (5)

For the treatment of PBO, in most cases extended antibiotic courses were used (median 49 d). The median (IQR) number of days from hospital admission to discharge for PBO assessment and treatment was 6.5 (3.8 d). Surgical approaches were varied. The bone biopsies were sent either by interventional radiologists or by orthopedic surgeons at the time of debridement. Descriptions of surgical interventions are included in Table 3.

Antibiotic regimens were determined by an infectious disease specialist and were individualized, and decisions were based on whether source control was achieved. The treatment regimens, durations, and outcomes are listed in Table 3.

**Table 3:** Individual patient characteristics, treatment, and outcomes. Note that n/a represents not applicable.

Patient ID	Underlying malignancy^a^	Fistula to pubic bone^b^	Culture results: bone or debrided tissue adjacent to bone	Antibiotics^c^	Duration of antibiotics (total intravenous plus oral) in weeks	Surgical intervention	Ambulation required assistance at last follow-up	Pain with ambulation at most recent follow-up	Time at last follow-up from PBO diagnosis (days)	Status at end of follow-up	Summary of last pelvic imaging findings at end of follow-up (X-ray, CT, or MRI)
1	GU	GU & GI	No growth (on antibiotics)	Etp + Dap; Lvx + Min; Crm	12	Bone biopsy	Yes	Yes	877	Dead	Progression of metastatic disease in pelvis; recurrent large rectovesical fistula. Chronic changes of pubic symphysis
2	GYN	GU	*Actinomyces* spp., *Aerococcus urinae*, *Bacteroides ovatus*	P/T, A/C	3	Resection of a portion of the pubic body, the inferior pubic ramus, portion of the ischium.	Yes	Yes	288	Dead	Progression of metastatic disease in pelvis, persistent fistula
3	GI, GU	No	No growth	T/S + A/C	1.8	Bone biopsy	Yes	Yes	416	Dead	Progression of metastatic disease in pelvis; erosion by tumor into left parasymphyseal region
4	GU	No	No growth	Cip	6	Bone biopsy	Yes	No	717	Dead	Healing of osteomyelitis changes of symphysis pubis; progression of disease
5	GU	GU	*Candida albicans*	Fluc	2	Curettage and symphysiotomy of the pubis	Yes	Yes	2195	Alive	Stable chronic osteomyelitis in the symphysis pubis
6	GU	No	Methicillin-resistant *Staphylococcus aureus*, *Enterococcus faecalis*	V + P/T; Dap; Cpt + Mtz; Amx	12	Bone biopsy	No	No	2430	Alive	No new suspicious osseous lesions
7	GI	No	No growth (on antibiotics)	P/T	6	Bone biopsy	Yes	Yes	576	Dead	Progression of metastatic disease in pelvis, pubic ramus fracture, fistula, abscess
8	GI	No	Not performed	T/S + A/C; P/T	24	n/a	Yes	Yes	133	Dead	Progression of metastatic disease, increased osseous destruction of right pubic ring
9	GYN	GU	Not performed	Cro + Mtz; Etp	> 1 year	n/a	Yes	Yes	1429	Dead	Persistent fistula; unchanged chronic sclerosis of bilateral pubis
10	GU	GU	No growth (on antibiotics)	Cro	8	Pubic bone debridement	No	No	1319	Alive	Healing pubic symphysis
11	GU	GU	*Pseudomonas aeruginosa*	Mer; Cip	4	Bone biopsy	No	No	1098	Alive	Stable findings of prior osteomyelitis pubis
12	GU	GU	*Serratia marcescens*	Etp; Cip	6	Pubic bone debridement, radical cystectomy, ileal conduit formation	No	No	1479	Alive	Stable fracture deformities of right superior and inferior pubic rami
13	GU	GU	No growth	P/T	8.4	Bone biopsy	No	No	991	Alive	Unchanged widening of pubic symphysis due to chronic osteomyelitis
14	GU	GU	Not performed	A/C	32	n/a	Yes	Yes	1199	Alive	Persistent fistula; chronic diastases and erosive changes of the pubis
15	GU	No	No growth	P/T	23	Bone biopsy	No	No	281	Alive	Increased pubic symphysis osteomyelitis with adjacent adductor abscesses
16	SAR	GU	*Enterobacter* cloacal complex (on antibiotics)	Crm; Mer; Etp; P/T	12	Pubic bone debridement, cystectomy, ileal conduit formation	Yes	No	942	Alive	Stable known sequelae of prior osteomyelitis
17	GU	GU & GI	Not performed	Etp; Mtz; P/T	12	Pubic bone debridement, cystectomy, ileal conduit formation, ileostomy, lower anterior resection	No	No	782	Dead	Chronic changes of the symphysis pubis
18	GU	GU	*Propionimicrobium lymphophilum*	Cfl	1	Pubic bone debridement, cystectomy	No	No	512	Alive	Chronic changes of the symphysis pubis

**Table 3:** Continued.

Patient ID	Underlying malignancy^a^	Fistula to pubic bone^b^	Culture results: bone or debrided tissue adjacent to bone	Antibiotics^c^	Duration of antibiotics (total intravenous plus oral) in weeks	Surgical intervention	Ambulation required assistance at last follow-up	Pain with ambulation at most recent follow-up	Time at last follow-up from PBO diagnosis (days)	Status at end of follow-up	Summary of last pelvic imaging findings at end of follow-up (X-ray, CT, or MRI)
19	GU	GU	*Aerococcus sanguinicola*, *Finegoldia magna*, *Gleimia europaea *(on antibiotics)	A/C	10	Radical resection of pubic symphysis and pubic rami, cystectomy, ileal conduit creation	No	No	398	Alive	Chronic changes in the symphysis pubis, chronic sclerosis in the posterior iliac bones and right sacrum
20	GU	GU & GI	Not performed	P/T Etp, Lvx, Mtz	8	Pubic bone debridement, laparoscopic colostomy, Hartmann's procedure, suprapubic catheter placement	No	No	643	Alive	Unchanged fistula. Less conspicuous erosive change along pubic bone
21	GU	GU & GI	Not performed	P/T, Etp	2	n/a	Yes	Yes	118	Dead	Increased metastatic disease in pelvis with necrotic tumor and abscess
22	GU	GU	Not performed	P/T	5.4	Pubic and pelvic bone debridement, radical cystectomy, ileal conduit creation	No	No	413	Alive	Post-surgical changes in symphysis pubis with residual simple fluid collection
23	GU	GU	*Escherichia coli* (on antibiotics)	Cro, T/S	4	Bone biopsy	No	No	306	Alive	Unchanged subchondral erosive changes at the symphysis pubis
24	GU	GU	*Lactobacillus* species, *Staphylococcus epidermidis*	Cfz, Dap, Etp	6	Pubic symphysis debridement, simple cystectomy, ileal conduit creation	No	No	2647	Alive	Unchanged ill-defined pubic bone sclerosis probably related to sequela of osteomyelitis
25	GU	No	Gram-positive cocci on gram stain (culture negative)	Dap	8	Bone biopsy	No	No	52	Alive	Resolved small retropubic fluid collection, decreased muscular edema
26	GU	No	*Escherichia coli*	T/S	8	Bone biopsy	Yes	No	195	Dead	Stable pubic symphysis erosions, progression of metastatic disease

Abbreviations: ^a^ Malignancy: GU = genitourinary; GYN = gynecological; GI = gastrointestinal; SAR = sarcoma. ^b^ Fistula: GU = genitourinary; GI = gastrointestinal. ^c^ Antibiotics: A/C = amoxicillin/clavulanate;Amx = amoxicillin; Cfl = cephalexin; Cfz = cefazolin; cip = ciprofloxacin; crm = cefuroxime; cro = ceftriaxone; Dap = daptomycin; Etp = ertapenem; Fluc = fluconazole; Lvx = levofloxacin; Mer = meropenem;Min = minocycline; Mtz = metronidazole; P/T = piperacillin/tazobactam; T/S = trimethoprim/sulfamethoxazole.

Patients were followed until death or date of last follow-up (median of 680 d; range: 52 to 2647 d). Outcomes were evaluated at the time of last follow-up. 10 out of 26 (38 %) patients had died at the end of follow-up.

Among the 15 patients (58 %) treated with antibiotics alone, 8/15 (53 %) had no pubic-bone-related pain at time of last follow-up, with 6/15 (40 %) walking without an assistive device at time of last follow-up. However, 9 of these 15 patients had died at the time of last follow-up, comprising 82 % of the total deaths in this case series. 8 of these 9 patients who died in this group had persistent fistulae, progression of malignant disease, and/or continued signs of abscesses and pubic bone destruction on the last imaging study (X-ray, CT, or MRI) before death.

There were 11 patients (42 %) who underwent pubic bone debridement, with 8/11 undergoing additional procedures such as cystectomy, ileal conduit creation, or colostomy creation for diversion of urine or gastrointestinal flora away from the pubic bone in addition to pubic bone debridement. Of the patients who had surgical interventions plus antibiotic treatment, 7/11 (63 %) had no pubic-bone-related pain at date of last follow-up, and 9/11 (81 %) were ambulating without an assistive device at time of last follow-up. 1 of these 11 patients had died at the time of last follow-up, comprising 10 % of the total deaths in this case series.

## 4 Discussion

Pubic bone osteomyelitis (PBO) is a rare complication that can occur after the treatment of cancers in the pelvis, either due to radiotherapy received in that region and/or pelvic or urologic surgical procedures previously performed to address a primary cancer. Despite the rarity of PBO as a complication following cancer treatment, it is important to promptly diagnose and employ a multidisciplinary approach to management to ensure the best outcomes. In our case series, patients diagnosed early with definitive surgical management that included source control and bone debridement had the best outcomes. These data support previous clinical data which also correlate better treatment outcomes with pubic bone debridement plus antibiotics (Gupta et al., 2015; Lavien et al., 2017; Nosé et al., 2020; Devlieger et al., 2021; Shu et al., 2021; Ambrosini et al., 2022; Hansen et al., 2022; Walach et al., 2024). Those who were frail, with advanced cancer and high risk for surgical debridement, still achieved some symptom palliation from antibiotic management alone.

A caveat to these findings is that the patients who did not undergo pubic bone debridement and instead were managed conservatively with antibiotics alone were likely to be in an overall sicker group, perhaps with poorer prognoses related to their malignancies, and this likely influenced decisions regarding surgery.

Among risk factors for PBO in the oncology setting, advanced age and males with GU malignancies, especially prostate cancer with a history of radiation and urologic procedural interventions, were the most common risk factors. Pelvic and pubic discomfort was the most frequent clinical presentation with lack of systemic symptoms. MRI was the most common diagnostic mortality for early changes. In patients with risk factors who present with pelvic discomfort, an early MRI may aid in timely diagnosis. Time from last cancer treatment or radiation therapy to onset of PBO symptoms may also be on the order of years; thus, patient symptoms should not be discounted as unrelated to prior cancer treatment regardless of how much time has passed since last cancer-directed therapy.

Points to consider for future studies include accounting for the optimal timing of antibiotic therapy or debridement in PBO pathogenesis and providing antibiotics in cases where no fistula or abscess is present on imaging. It is possible that providing antibiotics without surgical intervention if PBO is detected early, before the progression of bony destruction, may provide better outcomes compared to antibiotic treatment alone when antibiotics are started at a later point in the illness. (Anele et al., 2022; Burriss and Abualruz, 2023; Smeyers et al., 2024; Walach et al., 2024).

The strengths of our case series include the fact that it is the largest retrospective cohort from a single center to date focusing on real-world outcomes in cancer patients, along with the extended duration of follow-up. The limitations of this study include its retrospective nature and lack of standardized treatment approach and baseline patient risks that preclude any meaningful comparisons between conservative and surgical treatment approaches.

In conclusion, the management of PBO should utilize a multi-disciplinary approach that includes the oncologist, orthopedic surgeon, interventional radiologist, infectious disease specialist, and rehabilitation specialist. Any pubic-bone-related discomfort in patients with a history of pelvic malignancy – even long after treatment completion – should be promptly investigated with an MRI for early signs of PBO. Antibiotic therapy should be based on bone aspirate cultures (ideally obtained off antibiotic therapy), even for cases where aggressive debridement may not be possible. A combination of surgical debridement plus targeted antibiotic therapy offered the best outcomes.

## Data Availability

Data supporting the findings of this study are available from the corresponding authors upon reasonable request.
